# Thymoquinone Increases the Sensitivity of SW-480 Colon Cancer Cells to 5-Fluorouracil

**DOI:** 10.1155/2024/6231095

**Published:** 2024-07-09

**Authors:** Nima Zeinali, Vahid Mahmoudzadeh, Alireza Anarjani, Mohammad Ebrahimnejad, Bahman Yousefi, Amir Valizadeh

**Affiliations:** ^1^ Molecular Medicine Research Center Tabriz University of Medical Sciences, Tabriz, Iran; ^2^ Department of Clinical Biochemistry and Laboratory Medicine Faculty of Medicine Tabriz University of Medical Sciences, Tabriz, Iran; ^3^ Student Research Committee Tabriz University of Medical Sciences, Tabriz, Iran

**Keywords:** 5-Fluorouracil, *γ*-H2AX, apoptosis, colorectal cancer, drug resistance, thymoquinone

## Abstract

**Background:** Studies have concentrated on the therapeutic potential of thymoquinone (TQ), a natural polyphenol, in diverse malignancies, such as colorectal cancer. Nevertheless, the precise mechanisms of TQ-mediated anticancer properties are not yet fully elucidated.

**Objective:** The present study has been designed to scrutinize the impact of TQ on 5-fluorouracil (5-FU)–mediated apoptosis in SW-480 cells.

**Materials and Methods:** SW-480 cells were treated with TQ, 5-FU, and a combination of TQ + 5‐FU. MTT assay was employed to assess cell viability. Quantitative real-time polymerase chain reaction (qRT-PCR) was applied to evaluate apoptotic markers comprising Bcl-2, Bax, and caspase-9 expression levels. The *γ*-H2AX protein expression was assessed by western blotting, and Annexin V flow cytometry was implemented to determine the apoptosis rate.

**Results:** 5-FU significantly reversed the cell proliferation in a dose-dependent circumstance. The concurrent administration of TQ and 5-FU led to a substantial inhibition of cell growth in comparison to single treatments (*p* < 0.05). TQ also facilitated apoptosis via upregulating Bax and caspase-9 proapoptotic markers and suppressing antiapoptotic mediators, like Bcl-2. In addition, TQ augmented 5-FU-induced apoptosis in SW-480 cells. 5-FU, combined with TQ, increased the protein expression of *γ*-H2AX in SW-480 cells compared with groups treated with TQ and 5-FU alone.

**Conclusion:** The present study's findings unveil the significance of TQ as a potential therapeutic substance in colorectal cancer, particularly through enhancing 5-FU-induced apoptosis.

## 1. Introduction

Colorectal cancer stood out as the third most prevalent cancer type, with an escalating incidence rate, specifically in developing countries. Even though therapeutic methods, including chemo/radiotherapy, targeted therapy, and immunotherapy, have been widely applied in the treatment of colorectal cancer, results indicate a limited enhancement of overall survival in colorectal cancer patients. As one of the frequently used chemotherapeutics in colorectal malignancies, alone or in combination with other modalities, 5-fluorouracil (5-FU) is a specific inhibitor of thymidylate synthase, which interferes with the synthesis of pyrimidine thymidine, hence block DNA replication [1, 2]. Therefore, 5-FU induces apoptosis and suppresses cell proliferation in tumor cells. However, despite the favorable outcomes of 5-FU chemotherapy, over 90% of patients receiving 5-FU confront treatment failure [3]. A well-recognized reason contributing to the failure of combating colorectal cancer is the emergence of drug resistance during treatment with conventional chemotherapeutics. Aberrant expression patterns of drug transporters such as P-glycoprotein (P-gp), multidrug resistance protein 1 (*MRP1*), dysregulation of DNA repair process, cancer stem cells, resistance to cell death, and recently transcriptional disruption of miRNA are regarded as the most primary methods for development of chemotherapy-resistant cancer cell [4, 5]. Given that drug resistance is a notable obstacle in combating tumor cells, previous studies have concentrated on defining the exact molecular mechanisms responsible for drug resistance and exploring novel therapeutic molecules to target and reverse drug resistance.

Recently, increasing attention has been paid to the role of nutritional interventions in preventing and treating cancer [6, 7]. Among these, polyphenols have a special place. Polyphenols are bioactive compounds in large quantities in fruits, cereals, vegetables, grains, beans, and beverages like coffee and tea [8]. Despite antioxidant, antimicrobial, anti-inflammatory, antihypertensive, iron-chelating, and antiallergic abilities, polyphenols also possess a potent anticancer capacity and prevent cancer by strengthening the antioxidant system, detoxifying the body, and eliminating carcinogens and free radicals. In addition, various studies indicate the importance of these compounds in inhibiting cancer progression [9].

Thymoquinone (TQ), as a pivotal member of the polyphenol family, is predominantly present in a variety of fruits and vegetables, including apples, berries, wheat, and onions [10]. TQ exerts its anticancer properties through diverse mechanisms ranging from influencing cellular signaling to its capacity by suppressing enzymes crucial to central cellular processes [11, 12]. The broad-spectrum pharmacological and biochemical exhibited by TQ and its metabolites are attributed to the flavonol molecule's relative substitution of several functional groups [13]. Therefore, a large body of recent research has authenticated that TQ is an appropriate therapeutic candidate for a broad range of human malignancies, including colorectal cancer [14–16]. TQ exhibits anticancer activity and effects through various mechanisms, encompassing antioxidant function, inhibition of the proliferative process, and apoptosis stimulation [17, 18]. More interestingly, numerous studies have shown that concomitant use of some chemotherapeutic drugs with TQ increases their sensitivity to these drugs. Therefore, this study is aimed at evaluating the potentiating role of TQ in 5-FU-related apoptosis in SW-480 colon cancer cells.

## 2. Material and Methods

### 2.1. Cell Culture

Human SW-480 colorectal cancer cells were obtained from the Pasteur Institute Cell Bank of Iran (Tehran, Iran). Cells were cultured in RPMI-1640 medium containing 10% FBS and 100 penicillin/streptomycin per unit/milliliter and incubated at 37°C and 5% CO_2_. After the cells reached a density of 70%–80%, the cells were passaged by standard techniques.

### 2.2. Cell Viability Assay

3-(4,5-Dimethylthiazol-2-yl)-2,5-diphenyltetrazolium bromide (MTT; Sigma-Aldrich) assay was applied to evaluate cell viability in cells. MTT yellow tetrazolium is reduced by metabolically active cells, partly by the action of dehydrogenase enzymes, to produce reducing equivalents such as NADH and NADPH. The formed intracellular crystals of purple formazan can be dissolved and evaluated by spectrophotometric methods. For the MTT assay, 10,000 cells were cultured in 200 mL medium per well of a 96-well microplate. Twenty-four hours later, when the cell density reached 70%–60%, the cells were treated with different concentrations of 5-FU, TQ alone, and a combination of 5-FU and TQ for 48 h. Some wells were also considered as untreated control. After incubation, the supernatants of the cells were replaced with 200 *μ*L medium containing 50 *μ*L MTT solution (5 mg/mL in PBS) and then incubated in the dark for 37 h. After incubation, the mixture containing the MTT medium and solution was carefully removed, and 200 *μ*L DMSO was added to each well to dissolve the crystals. Finally, the absorption of each well was measured at 570 nm using a microplate reader (Biotek, ELx800, USA) 15 min after shaking.

### 2.3. Combination Index (CI) Analysis

The CI is a metric used to evaluate the cytotoxic effects of combining two drugs. It is calculated using the following formula: CI = (*D*1/*Dx*1) + (*D*2/*Dx*2) + *α*(*D*1*D*2/*Dx*1*Dx*2), where *D*1 and *D*2 are the concentrations of the two drugs in the combination, *Dx*1 and *Dx*2 are the concentrations of each drug alone that give 50% reduction in cell viability, and *α* is 0 or 1 depending on whether the drugs are mutually exclusive or nonexclusive.

A CI < 1 indicates a synergistic interaction between the drugs, CI = 1 is an additive effect, and CI > 1 represents an antagonistic effect.

### 2.4. RNA Isolation and Real-Time Polymerase Chain Reaction (PCR)

Total RNA was isolated from the cells via TRIzol reagent based on the manufacturer's guidelines. Then, complementary DNA (cDNA) templates were synthesized using cDNA synthesis kit. Finally, synthesized cDNA was subjected to quantitative real-time PCR (qRT-PCR) in duplicate using the SYBR Green master mix and Mic qPCR Cycler. *β*-Actin was used as the reference gene for all samples to normalize the mRNA expression level. The results of qRT-PCR were calculated using the 2^−*ΔΔ*C^_T_ method. Sequences of primers used to amplify Bax, Bcl-2, caspase-9, and *β*-actin are listed in Table 1.

### 2.5. Flowcytometric Detection of Apoptosis

SW-480 cells (300,000 cells per well) were cultured in a 6-cell plate to a density of 70%. The cells were then divided into 4 groups: (1) control group, (2) group treated with 5-FU, (3) the group treated with TQ, and (4) group treated with TQ and 5-FU. Forty-eight hours later, the cells were trypsinized and washed twice with PBS and once with binding buffer after centrifugation. Cell concentrations were adjusted to 500,000 to 600,000 cells by 100 *μ*L binding buffer per well. Then, 5 *μ*L Annexin V conjugated with fluorochrome was added to each sample. The cells were incubated for 10–15 min at room temperature. In the next step, the cells were washed with binding buffer and, this time, dissolved in 200 *μ*L binding buffer. Then, 5 *μ*L of propium iodide staining solution was added. The extent of cell apoptosis was detected by flow cytometry.

### 2.6. Western Blotting

SW-480 cells are cultured in plate 6 cells at RPMI with 10% FBS. After the cells were treated, the protein was extracted. The total amount of 20 *μ*L of the extracted protein was then boiled with a sample buffer mixed for 5 min. The samples were then electrophoresed on polyacrylamide gel at 100 volts, and the proteins were transferred to the PVDF membrane in the Western Transfer buffer using a Bio-Rad Semi-Dry Transfer Cell Trans-Blot SD. The membrane was blocked using a wash buffer containing 5% skim milk powder for 1 h at room temperature and then washed three times for 5 min with a wash buffer. The membrane was then incubated overnight with the primary antibody against *γ*-H2AX at 4°C. The membrane was washed three times with a wash buffer for 5 min and then incubated with HRP-conjugated mouse IgG secondary antibody for 2 h at room temperature. Immunoblots were visualized using an enhanced chemiluminescence ECL detection reagent. For detection, the membrane was incubated in ECL kit solutions for 5 min according to company protocol. A large-range protein marker was used as a molecular weight marker.

### 2.7. Statistical Analysis

Results were shown as mean ± SD in at least three separate experiments. Statistical analysis was performed using SPSS software and GraphPad Prism V6 through *t*-test or ANOVA, and a *p* value of less than 0.05 was considered statistically significant.

## 3. Results

### 3.1. The Effect of 5-FU on the Proliferation of SW-480 Cell Line

**Figure 1:**
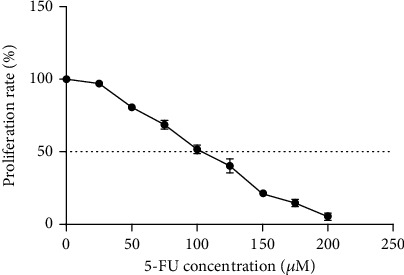
The effect of 5-fluorouracil on SW-480 cell line proliferation rate. The results are shown as mean ± SD after three repetitions of experiments.

The cytotoxic effects of 5-FU on SW-480 cells were evaluated using MTT assay. After treatment of cells with different concentrations of 5-FU and 48-h incubation, the amount of cytotoxicity was calculated using the formula (Figure 1). As shown in Figure 1, the cytotoxicity of 5-FU increased with increasing drug concentration. In the SW-480 cell line, the IC50 of 5-FU was 100 ± 1 *μ*M after cell treatment for 48 h.

### 3.2. The Effect of TQ Use on the Proliferation Rate of SW-480 Cell Line

**Figure 2:**
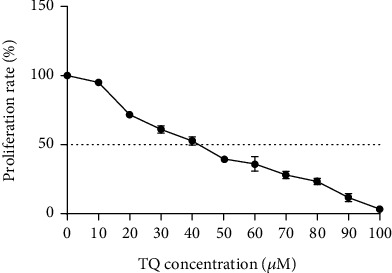
The effect of thymoquinone on SW-480 cell line proliferation rate. The results are shown as mean ± SD after three repetitions of experiments.

The proliferation of SW-480 cells in the presence of TQ was also examined using an MTT assay. As shown in Figure 2, compared to the control group, cell treatment with TQ significantly reduced cell proliferation after 48 h in a dose-dependent manner. The IC50 value for TQ is 41 ± 1 *μ*M.

### 3.3. The Effect of Using 5-FU and TQ on the Proliferation of SW-480 Cell Line

**Figure 3:**
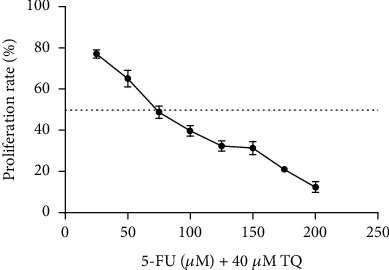
Effect of 5-fluorouracil and thymoquinone combination on SW-480 cell line proliferation. The results are shown as mean ± SD after three repetitions of experiments.

In addition, the combined effects of 5-FU and TQ on cell proliferation were also evaluated in this study. Combination therapy further reduced cell proliferation compared with 5-FU or TQ monotherapy (Figure 3). Combining concentrations less than 75 *μ*M 5-FU with 40 *μ*M, TQ could not induce 50% cytotoxicity in cells (CI = 0.9). Concentrations above 75 *μ*M showed a synergistic interaction in SW-480 cells after 48 h of incubation (Figure 3). In other words, the combination of 5-FU and TQ reduced the IC50 values of 5-FU from 100 to 75 *μ*M in this cell line. In fact, combining 5-FU with TQ increases the cytotoxicity of 5-FU.

### 3.4. The Effect of 5-FU and TQ Combination on SW-480 Cell Line Apoptosis

First, the expression of apoptotic factors was examined. To investigate changes in apoptotic factors including Bax, Bcl-2, and caspase-9 in different groups treated with 5-FU, TQ, and a combination of the two, cells were first divided into four groups:

• Group 1: SW-480 cells without any treatment and as a control group
• Group 2: SW-480 cells treated with 100 *μ*M 5-FU
• Group 3: SW-480 cells treated with 40 *μ*M TQ
• Group 4: SW-480 cells treated with 100 *μ*M 5-FU and 40 *μ*M TQ (5‐FU + TQ)

After cell treatment, RNA was extracted from the cells, and then, cDNA was synthesized. The expression of all three specific genes that are markers of apoptosis was measured using specific primers.

**Figure 4:**
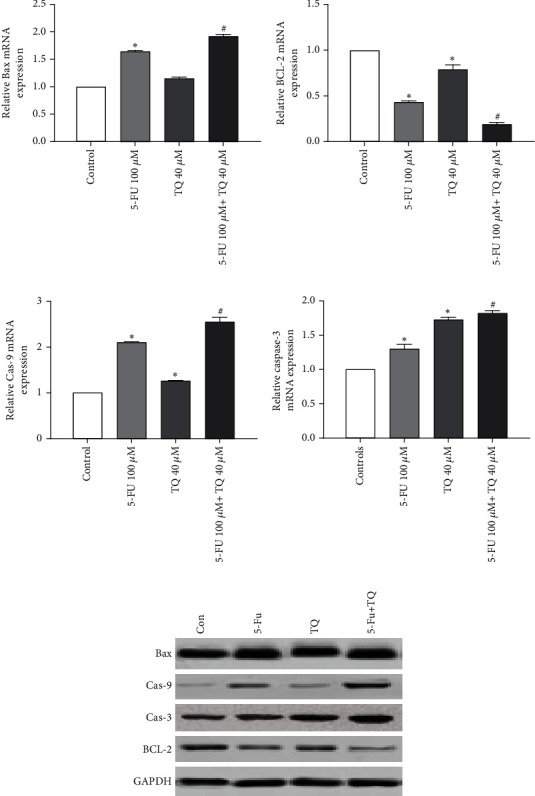
(a–d) The mRNA expression levels of Bax, BCL-2, caspase-3, and caspase-9 in the treated group. (e) Bax, BCL-2, caspase-3, and caspase-9 protein expression levels in the treated group. The results are shown as mean ± SD after three repetitions of experiments.  ^∗^*P* < 0.05 vs control group, ^#^*P* < 0.05 vs 5-FU group.

Bax is a proapoptotic gene that promotes apoptosis. As shown in Figure 4(a), treatment of cells with 5-FU and TQ alone increased Bax expression in cells compared with the control group (*p* < 0.05). 5-FU and TQ in combination with each other have a greater effect on increasing the expression of this gene in the SW-480 cell line (*p* < 0.05).

Bcl-2, unlike Bax, is an antiapoptotic gene that prevents the progression of apoptosis. In our study, the expression level of Bcl-2 in cells treated with 5-FU and TQ alone was significantly lower than the control group (*p* < 0.05; Figure 4(b)). On the other hand, the expression of this antiapoptotic gene in cells treated with 5-FU and TQ was much lower than in the treated groups alone (*p* < 0.05).

The next gene to be studied in our study is caspase-9, which is an important proapoptotic gene. In this study, treatment of cells with 5-FU and TQ alone increased the expression of caspase-9 in cells compared to the control group (*p* < 0.05; Figure 4(c)). 5-FU and TQ, combined with each other, have a greater effect on increasing the expression of this gene in the SW-480 cell line (*p* < 0.05). Similar results were also found for caspase-3, another key proapoptosis gene. The results showed that combining 5-FU and TQ led to potent induction in the expression levels of caspase-3 (*p* < 0.05; Figure 4(d)).

### 3.5. The Effect of 5-FU and TQ on the Apoptotic Response by Flow Cytometry of SW-480 Cell Line

**Figure 5:**
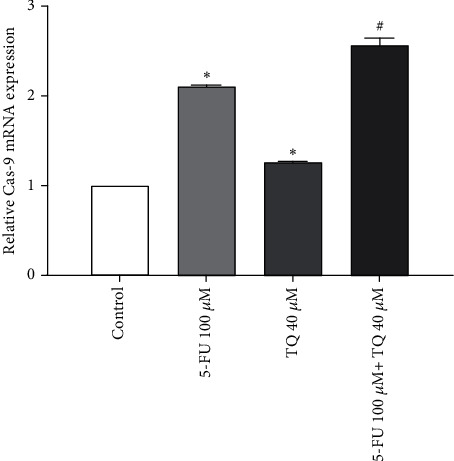
Effect of thymoquinone and 5-fluorouracil on the apoptosis rate in the treated group. The results are shown as mean ± SD after three repetitions of experiments. ^∗^*P* < 0.05 vs control group, ^#^*P* < 0.05 vs 5-FU group.

In addition, the number of apoptotic cells in the study groups was also measured by flow cytometry. As shown in Figure 5, the results show that the rate of final apoptotic response in cells treated with 5-FU and TQ is 65% and 18%, respectively. The apoptosis rate in untreated cells as a control group is 5%. On the other hand, the combined treatment of cells with 5-FU and TQ as a synergist has a stronger effect on inducing apoptosis compared to monotreatment with any of the drugs. The apoptosis rate in cells treated with both drugs is 80% compared with 5% in control cells.

In general, 5-FU and TQ increase proapoptotic gene expression and decrease antiapoptotic gene expression. However, combining the two has a more substantial effect on promoting apoptosis in the SW-480 colorectal cancer cell line. In other words, TQ increases the sensitivity of SW-480 cells to apoptosis induced by 5-FU.

### 3.6. The Effect of 5-FU and TQ on the Expression of *γ*-H2AX SW-480 Cell Line

**Figure 6:**
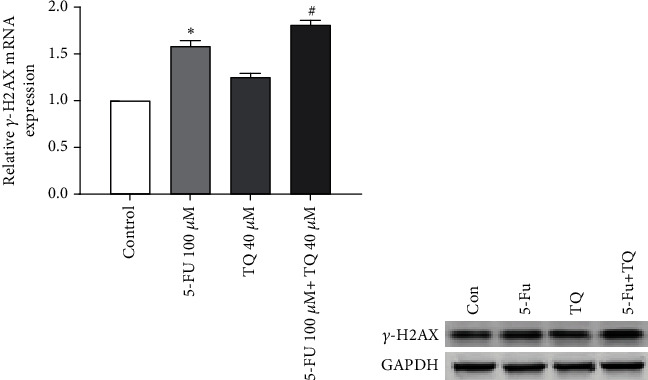
(a, b) The changes in expression of *γ*-H2AX in mRNA and protein levels in the treated group. The results are shown as mean ± SD after three repetitions of experiments. ^∗^*P* < 0.05 vs control group, ^#^*P* < 0.05 vs 5-FU group.

*γ*-H2AX is an important marker of DNA damage in cells. As a result, the expression of this protein in the treated groups was measured using western blotting. The amount of *γ*-H2AX protein in cells treated with 5-FU and TQ alone was significantly higher than in the control group (*p* < 0.05; Figures 6(a) and 6(b)). On the other hand, the expression of this protein in cells treated with 5-FU and TQ was very high compared to the treated groups alone (*p* < 0.05).

## 4. Discussion

Colorectal cancer accounts for one of the leading causes of cancer-related death globally [19]. Despite recent progress in both diagnosis and therapy of this disease, the general prognosis of colorectal cancer remains unsatisfactory [20]. The therapeutic approach commonly involves neoadjuvant chemotherapy preceding surgical removal and subsequent adjuvant chemotherapy [21]. 5-FU, as one the most potent chemotherapeutic agents in colorectal cancer, inhibits thymidylate synthase and incorporates its metabolites into RNA and DNA to generate anticancer properties [2]. However, diminished survival rates of metastatic patients and suboptimal efficacy of chemo drugs in advanced stages of disease are among the limitations that constrain existing therapeutic modalities.

Furthermore, some patients suffer chemotherapy resistance, which, in some instances, is elucidated as tumor recurrence or progression [22–24]. It is essential to discern between two types of resistance: acquired and internal resistance [25–27]. Internal resistance is inherent as resistant cells exist within the tumor before initiating therapy. Upon the administration of chemotherapy, sensitive cells succumb to the drug's cytotoxic effects. Even so, in the presence of chemotherapeutic medicines, tumor-resistant cells that trigger numerous signaling pathways or exhibit pre-existing genetic alterations might increase [5].

On the other hand, acquired resistance emerges post-treatment and is mediated by drugs. During treatment, the anticancer impact of the chemo drugs is gradually palliated. Indeed, resistant cancer cells undergo mechanisms that alter the expression of drug targets or transport protein mutations, stimulate proto-oncogenes, and modify the tumor microenvironment [28]. As a result, these modifications enable tumor cells to withstand chemotherapy. Therefore, resistance arises from genomic instability, where resistant clones are chosen following chemotherapy. Operating system cells' unique molecules, protein regulators, and signaling cascades are responsible for both forms of resistance [3]. Thus, more potent and urgent therapies are required to overcome it. Deciphering the molecular mechanisms and a better knowledge of conventional chemotherapy resistance is essential to provide novel strategies aiming to develop specific personal treatments and ultimately enhance overall survival [29].

A great deal of evidence (mostly preclinical research) indicates that TQ, by itself or combined with other common chemotherapy medications, can substantially impede cancer progression and able the tumor burden in a variety of malignancies by influencing drugs or altering tumor pathways.

A study by Pazhouhi et al. suggested that TQ synergistically potentiates the anticancer ability of temozolomide (TMZ) in the U87MG glioblastoma cell line by preventing autophagy [30]. In line with the recent study, Khazaei and Pazhouhi showed that apoptotic cell death was correlated with combination therapy of TQ and TMZ in glioblastoma cells [31]. Similar findings in another study revealed that TQ inhibits cell growth and induces cell cycle arrest, DNA damage, and apoptosis in glioblastoma tumor cells [32]. In fact, TQ induced telomere shortening in GBM cells via reversing telomerase function. This effect is notably heightened in GBM cells that express DNA-PKcs in vivo [31]. Another study on the rate of colorectal tumor models reported that TQ significantly reduces tumor growth and strengthens the impact of 5-FU on cancer cells [33]. In this research, azoxethane (AOM) was applied to induce colorectal neoplasia, and the combined treatment of 5-FU/TQ further alleviated AOM-mediated colorectal tumors and large ectopic crypt foci compared with both agents alone [33]. Additionally, it has been shown that elevated ROS production and induction of concomitant DNA damage in human colon tumor cells is mediated with a new hybrid of TQ and artemisinin [34]. Except for colorectal cancer, the ability of TQ as a potent combination agent with 5-FU got bolder when Lei et al. found that TQ can promote the sensitivity of gastric cancer cells to 5-FU by facilitating apoptosis and inhibiting proliferation [35]. Overall, the findings of our investigation are congruent and in line with earlier research. Our study showed that the combination of 5-FU and TQ decreased the IC50 levels of 5-FU, or in other words, increased the cytotoxicity of 5-FU in SW-480 colorectal cancer cell lines [36, 37]. 5-FU and TQ increase the protein expression of proapoptotic genes, diminish the antiapoptotic protein level, and upregulate the *γ*-H2AX protein as the main DNA damage marker. As a result, the combination of these two anticancer agents exerts a more potent effect on promoting apoptosis in SW-480 cells. In other words, TQ enhances cell sensitivity to 5-FU in colorectal cancer cells.

## 5. Conclusion

As mentioned above, preclinical research outcomes advocate for the consideration of TQ in clinical settings. Researchers now possess substantial knowledge regarding TQ's molecular anticancer activity, novel drug delivery methods, drug toxicity, bioavailability, and pharmacokinetics. In conclusion, our results underscore the robust potential of using apoptosis targeting as a treatment strategy to increase the 5-FU response in colorectal cancer. Nevertheless, selecting a complementary drug to be combined with 5-FU is paramount, as our research has demonstrated that TQ exhibits significant promise as a therapeutic agent in preclinical tumor models. Since TQ has emerged as a potential option for upcoming clinical trials in cancer patients, these findings show its positive effects on increasing the susceptibility of colorectal cancer cells to conventional chemotherapy drugs.

## Figures and Tables

**Table 1:** List of primers.

**Gene**	**Forward primer (5**′**–3**′**)**	**Reverse primer (5**′**–3**′**)**
Bax	GGTTGTCGCCCTTTTCTA	CGGAGGAAGTCCAATGTC
Bcl-2	GATGTGATGCCTCTGCGAAG	CATGCTGATGTCTCTGGAATCT
Caspase-9	GTGGAACTGACGATGATATGGC	CGCAAAGTGACTGGATGAACC
*β*-Actin	TCGTGCGTGACATTAAGGAG	AGGAAGGAAGGCTGGAAGAG

## Data Availability

The authors declare that all data supporting the findings of this study are available in the article and can be provided by the corresponding authors on reasonable request.
